# One sport, one family: negotiating inclusion, identity, and difference in national sports organizations

**DOI:** 10.3389/fspor.2025.1641914

**Published:** 2025-11-25

**Authors:** Kim Wickman, Malin Andersson

**Affiliations:** 1 Department of Education, Faculty of Social Sciences, Umeå University, Umeå, Sweden; 2 Department of Education, Umea Universitet, Umeå, Sweden; 3 Department of Education, Umeå University, Umeå, Sweden

**Keywords:** equity, inclusion, ability, disability, ingroup/outgroup, sport, parasport, power relations

## Abstract

**Introduction:**

Efforts to advance equity, diversity, and inclusion in sport aim to challenge systemic discrimination and promote equitable participation. In Sweden, Strategy 2030 represents a national initiative to integrate parasport into mainstream sport structures by embedding inclusive practices across all national sports organizations (NSOs). This study examines how key stakeholders within Swedish NSOs construct, interpret, and implement inclusion in this policy context. Guided by social identity theory (SIT), the study explores how processes of categorization, identification, and comparison influence ingroup/outgroup boundaries and shape inclusion practices.

**Methods:**

The study draws on qualitative, semi-structured interviews with 12 stakeholders formally mandated to implement inclusion within Swedish NSOs. Data were analyzed using reflexive thematic analysis to identify recurring patterns in how inclusion is understood, negotiated, and enacted within organizational settings.

**Results:**

The analysis revealed persistent tensions between formal commitments to inclusion and structural conditions that reproduce hierarchies of ability and belonging. Participants expressed strong normative support for inclusive values but described challenges in translating these values into practice within entrenched organizational cultures marked by ableist norms and resource disparities. Stakeholders also negotiated identity boundaries beyond the abled/disabled binary, indicating that inclusion efforts are shaped by intersecting dimensions of identity.

**Discussion:**

The findings illuminate how identity processes, power relations, and normative assumptions influence the implementation of inclusion policy at the organizational level. Despite policy ambitions, structural and cultural constraints limit transformative change. The study contributes to understanding the complexities of fostering inclusion in sport governance and underscores the need for more equitable, reflexive, and context-sensitive policy frameworks.

## Introduction

Efforts focused on equity, diversity, and inclusion seek to challenge inequality and discrimination by promoting fairness, respect, and equitable treatment for all individuals and groups. These initiatives aim to foster more just and inclusive institutions and societies (1). The foundation of such principles can be traced to the Universal Declaration of Human Rights. Adopted by the United Nations (UN) General Assembly in 1948, the Declaration outlines the essential rights and freedoms inherent to human dignity. Similarly, equal opportunities for persons with disabilities (PwD) in sports participation are a fundamental principle of the United Nations Convention on the Rights of Persons with Disabilities (2). According to the World Health Organization (WHO) (3), PwDs constitute the largest minority group globally. As of 2023, approximately one billion individuals—or around 16% of the global population—were living with a disability. This number is projected to increase as populations age and more individuals develop chronic health conditions that lead to disability. Although their numbers, PwD often encounter discrimination, driven largely by negative societal attitudes toward disability (4).

**Table 1:** Overview of respondents and national sport organizations (NSOs).

Sport (NSO)	*n*	Type	Inclusion stage^a^	Development officer role^b^
Floorball	4	Team	Early stage	Yes
Athletics	3	Individual	Early stage	Yes
Swimming	1	Individual	Further advanced	Yes
Table Tennis	2	Individual	Further advanced	Yes
Football	2	Team	Early stage	Yes

NSO, national sport organization.

a Early stage = NSO had initiated but not yet progressed far in the inclusion process; Further advanced = NSO had progressed further.

b All respondents held national-level development officer (or equivalent) responsibilities, including leading the “responsibility transfer”, coordinating a steering group, and managing internal/external communication and organizational development related to inclusion.

Despite these international commitments and the growing policy focus on equality, equity, diversity, and inclusion, the implementation of inclusive principles within sports remains uneven. Legal recognition of the rights of PwDs has not automatically translated into equitable participation in practice. As a result, it is essential to critically examine how these ideals materialize—or fail to materialize—in everyday sports settings.

The reality of sports practice continues to reveal persistent inequities, as PwDs often lack access to the tailored support and conditions necessary for full and fair participation alongside theirpeers in mainstream sports (5–7). Rather than being the result of personal choice, PwDs' opportunities to engage in sports are frequently constrained by environmental factors (e.g., physical and organizational barriers) and attitudinal obstacles (e.g., social perceptions) shaped by broader social contexts (6, 8) and power relations (9).

The growing recognition of the equal rights of PwDs in sports has led to increasing efforts to integrate parasports within mainstream sports systems, rather than maintaining separate parasport-specific structures. This process, commonly referred to as *mainstreaming*, involves the organizational and structural reconfiguration of parasport by transferring responsibility from specialized parasport federations to national sports organizations (NSOs) (10, 11). Several countries have undertaken mainstreaming efforts to promote inclusive sports environments, including England and Wales (11), France (12), Norway (13, 14), Switzerland (15), Australia (10), Canada (16), the Netherlands (17), and Sweden (18, 19).

Sweden is currently undergoing a large-scale transition that aims to transfer all parasports from the Swedish Parasport Federation to their mainstream counterparts (20). This transition aligns with Strategy 2030, a national policy initiative that aspires to ensure inclusion “leads to full participation and creates even better conditions for athletes, leaders, and clubs” (20). The Swedish Sports Confederation, the country's largest sports governing body, plays a central role in this organizational shift. It oversees nearly 20,000 nonprofit local sports clubs and implements inclusive sports policies (21).

Although mainstreaming is intended to promote inclusion, it has faced significant challenges in practice (22). For example, Van Lindert et al. (23) observed that no European country currently collaborates in a systematic or institutional way with sports clubs, sports federations, disability advocacy groups, and the state. This lack of coordination results in fragmented, opaque, and often confusing parasport systems that hinder the development and implementation of inclusive practices. Furthermore, studies suggest that rather than fostering the integration of values between mainstream and parasport cultures, mainstreaming has often served as a rationalization or efficiency-driven restructuring of sports organizations (11). In Canada, for example, evidence suggests that the integrated parasport model has failed to achieve its original inclusion goals, leaving para-athletes marginalized within the mainstream system (24). Similarly, mainstreaming has been criticized as a rhetorical tool with limited tangible effects (25) and has even been linked to assimilationist risks, where para-athletes are expected to conform to mainstream standards rather than having their specific needs fully accommodated (14).

One way to explain these persistent barriers is through the lens of social identity theory (SIT), which posits that individuals derive their sense of self through group membership (26, 27). According to SIT, people engage in social comparison to distinguish their identities from outgroups, typically in ways that elevate their own status. Applied to ableism—an ideological system embedded in societal structures and norms that prioritize able-bodiedness and frame disability as a deviation from the norm (28)—this means that non-disabled individuals may reinforce their self-esteem by forming and maintaining negative beliefs about PwD. SIT thus offers a psychological explanation for the social motivations underlying ableism. From this perspective, such barriers also reflect stereotypical assumptions about what “real” sport is and should be about (29, 30).

Ableism also continues to shape how sports organizations interpret and implement inclusive policies, often reinforcing preferences for able-bodied athleticism and shaping expectations regarding performance and participation (31, 32). While interest in mainstreaming has increased since the adoption of the United Nations Convention on the Rights of Persons with Disabilities (2), research on how it is implemented in practice remains limited (33). Most previous studies have examined mainstreaming from a governance-level perspective, focusing primarily on policy and organizational structures (11, 14). However, mainstreaming is not only an institutional process; normative understandings of what constitutes “real” sport also shape how inclusion is perceived and practiced—ultimately influencing who is granted access and under what conditions (29).

In the Swedish context, research has highlighted a significant gap between inclusion policy and practice (18, 19, 22). For example, Nordlund et al. (22) found that while the inclusion of para-athletes in mainstream sports is broadly supported in principle, its implementation is hindered by structural and practical challenges. Although inclusion is valued for promoting shared participation and mutual recognition between para-athletes and their non-disabled peers, stakeholders cited limited financial resources, inaccessible facilities, and a lack of adapted equipment as major obstacles. These findings reveal a deeper tension: Inclusion is often understood as integration into existing structures rather than as a transformation of those structures. This interpretation risks reinforcing ableist assumptions and failing to address the specific needs of para-athletes.

Andersson et al. (18) examined both enabling and constraining conditions for mainstreaming within Swedish sports clubs using frame factor theory. This approach considers how structural, functional, and methodological elements shape inclusion efforts. The study shows that inclusion is often influenced by individual leaders' interpretations and willingness to adapt, underscoring the relational and interpersonal dimensions that extend beyond policy directives. These findings call for a more critical and context-sensitive approach to inclusion—one that addresses environmental barriers, challenges normative conceptions of sport, and ensures that inclusion fosters equity rather than assimilation. To successfully realize inclusive efforts, it is essential to move the focus from the para-athlete to the broader sport environment and a critical examination of its accessibility.

To effectively bridge the gap between policy and practice, it is essential to explore how stakeholders responsible for implementing inclusion understand and carry out their roles within the structural and cultural frameworks of their sports organizations.

When analyzing barriers to participation, it is also critical to consider how disability intersects with other aspects of social identity. These dimensions do not exist in isolation; rather, they overlap and interact in ways that can compound marginalization. In this study, the concept of intersectionality offers a valuable analytical lens for understanding how multiple forms of disadvantage converge in people's lived experiences (34). For instance, promoting equality without accounting for equity risks reinforcing existing disparities, whereas an equity-informed approach actively seeks to dismantle them. An intersectional analysis reveals the need for institutions and policymakers to critically examine whether their diversity and inclusion efforts meaningfully incorporate disability—or whether disability remains excluded from broader equity agendas (35).

## Aim

This study aims to explore how key stakeholders within NSOs construct, perceive, and navigate social identities in the context of implementing inclusion across parasports and mainstream sports. Grounded in SIT, the research investigates how social categorization, identification, and comparison processes contribute to the emergence of ingroup/outgroup dynamics, particularly those related to able-bodied and disabled identities, and how these dynamics shape inclusion practices, intergroup relations, and the pursuit of social change. The study also seeks to understand how overlapping identity constellations beyond the able/disabled binary influence stakeholders' strategies for fostering inclusive environments without reinforcing status hierarchies or ingroup bias.

The study addresses the following research questions: (RQs):

RQ 1: How do key stakeholders within NSOs construct and understand social identities in relation to mainstream sports and parasports during the implementation of inclusion policies?
RQ 2: In what ways do processes of social categorization, identification, and comparison reinforce ingroup/outgroup dynamics within NSOs, and how do these dynamics influence inclusion practices and decision making?
RQ 3: How do stakeholders navigate intersecting social identities beyond the able/disabled binary?

## Theoretical framework: social identity theory (SIT) and ableism

SIT, developed by Tajfel and Turner (26), provides a valuable lens for understanding how individuals derive a sense of self through group membership. Drawing on Festinger's (36) social comparison theory, SIT proposes that people categorize themselves and others into ingroups and outgroups, identify with specific groups, and engage in favorable comparisons to maintain a positive self-concept (37). For example, functional perfection is positioned as both taken for granted and idealized, and individuals are judged based on how well their bodily or cognitive functions align with these expectations. In this way, people are categorized as either normative or non-normative (29).

According to Campbell (28), individuals who are unable to conform to the norm of compulsory able-bodiedness are subject to negative attitudes, exclusion through material and social inaccessibility, and often internalize the ableist power structures—viewing their own functionality and sense of self as deviant or inferior. In sports, where group distinctions based on ability are often emphasized, such processes can reinforce exclusionary dynamics (29).

Recent research has demonstrated the value of SIT in examining how marginalized individuals experience sports environments. In a systematic review of 88 studies, Noro and Mourão (38) synthesized the application of SIT in research on LGBTQIA+ individuals in sports. Their analysis revealed that SIT provides a robust framework for understanding both personal processes (e.g., identity construction, ingroup affection, and belonging) and intergroup dynamics (e.g., social comparison, discrimination, and intergroup mobility). The findings highlight how group-based identities shape access, inclusion, and lived experiences in sports, particularly for those facing systemic barriers and stigma. Similarly, Koo et al. (39) critically evaluated the limitations of SIT and role identity theory (RIT) in explaining the full complexity of sports fan behavior. While both theories provide insights into group affiliation and role-based behavior, the authors argue that they overlook the deep emotional, relational, and self-sacrificial aspects of sports fandom. To address this gap, they proposed identity fusion theory (IFT), which posits that personal and group identities can become fused into a single, powerful emotional bond. According to the authors, IFT not only complements but also extends SIT by emphasizing the emotional and motivational dimensions of identification, thereby offering a more nuanced and comprehensive framework for understanding the dynamics of sports fandom. These theoretical contributions point to the continued relevance of SIT while also highlighting the need to integrate other perspectives for a more comprehensive analysis of identity and inclusion in sport.

Building on these insights, the present study applies SIT to the context of parasport and inclusion, with particular attention to how disability intersects with broader notions of identity and belonging. In doing so, this research expands the relevance of SIT within sport studies by examining how stakeholders navigate inclusion in the context of ableist sport structures, responding to ongoing calls for a more intersectional and critically engaged application of the theory.

To better understand the power relations embedded in these processes, SIT can be complemented by a structural analysis of ableism—defined as a belief system and set of practices that privilege able-bodied norms while marginalizing disability (28). While SIT explains *how* group identities are formed and maintained, ableism reveals *why* certain group boundaries carry more social weight and lead to deeper inequalities. For example, ableist assumptions—such as defining “real” sports through able-bodied performance standards (29, 30)—are not neutral. They reflect and reproduce dominant norms that regulate access, visibility, and legitimacy in sports.

In this sense, ableism strengthens and extends SIT by emphasizing that social identity formation is not only a psychological process but also a process embedded within and shaped by systematic oppression. Where SIT might describe *how* individuals come to see para-athletes as an outgroup, ableism explains *why* that group is devalued in the first place and how institutional structures sustain that devaluation. This theoretical combination is especially relevant to parasport mainstreaming efforts. SIT helps explain the shifting social identities and intergroup dynamics that arise as parasport is integrated into NSOs. At the same time, ableism draws attention to how dominant norms and institutional frameworks may resist or distort these inclusion efforts, ensuring that structural inequalities persist, even after formal integration.

Furthermore, SIT's concepts of identity threats and ingroup protectionism help explain the subtle exclusion mechanisms that are activated during institutional change. For example, stakeholders may resist inclusion if they perceive it as threatening the perceived competence or coherence of their group. However, without considering ableism, such resistance may be interpreted merely as intergroup tension rather than as a defense of entrenched privilege. Integrating both frameworks allows for a more comprehensive analysis of how inclusion is not only enacted but also constrained. Finally, this study draws on the social identity approach to disability (4), which integrates SIT's focus on group dynamics with the structural critiques of critical disability studies. Labeling theory (40) and stereotype attribution (41) further contribute to understanding how perceived deviations from able-bodied norms contribute to the stigmatization and marginalization of disabled athletes. Together, these perspectives enable a deeper understanding of how inclusion in sports is negotiated, resisted, and sometimes subverted, both interpersonally and structurally.

## Ethical considerations and research context

Ethical considerations were rigorously addressed throughout the study. The project was designed and conducted in accordance with the Swedish Research Council's ethical standards for research in the humanities and social sciences. It forms part of a larger research project approved by the Swedish Ethical Review Authority (Dnr 2020-05067) and adheres to the Swedish Research Council's guidelines for good research practice. The project also complies with international ethical standards, including the European Union's General Data Protection Regulation (GDPR 2016/679) (42).

All participants provided informed consent, either in writing or orally, in accordance with ethical approval guidelines (43). Participants were fully informed about the study's purpose, design, and procedures. They were assured that all empirical data would be handled confidentially, that participation was voluntary, and that appropriate measures had been implemented to minimize the risk of identification (43). To ensure confidentiality and data protection, all participants' names were coded and anonymized prior to data analysis. Any other personal names or identifying information mentioned during the interviews were also anonymized. The interview settings were chosen carefully to safeguard participant privacy and confidentiality. In the presentation of the findings, identifying details were removed, and pseudonyms (e.g., 501A1 through 512A1) were used to represent the participants. The study adhered to the principle of voluntary participation, which allows participants the right to withdraw from a study at any point without penalty or consequence. As mentioned previously, both the research design and data collection procedures were reviewed to ensure full compliance with ethical guidelines aimed at protecting participants' rights and well-being. Throughout the study, particular attention was paid to respecting participants' autonomy, privacy, and dignity. Every effort was made to minimize potential harm or discomfort, especially when discussing sensitive topics, such as interpersonal conflicts. Ethical integrity was a guiding principle at all stages of the research process.

## Data collection and sample

Participants were selected based on their relevant expertise and potential influence on decision-making related to the study's focus. It was therefore essential to include key actors with formal authority and a mandate to guide the Swedish Sports Confederation toward its inclusion goals. Consequently, stakeholders from five NSOs, floorball (four respondents), athletics (three), swimming (one), table tennis (two), and football (two) were chosen (Table 1). All held national-level authority within their respective federations.

At the time of the interviews, all respondents were responsible for overseeing the so-called “responsibility transfer” of specific sports as part of the national inclusion effort. According to the Swedish Parasport Federation, parasport should be a natural and integrated component of the broader sports movement—not a separate system. Consequently, the goal is to transfer responsibility for certain sports currently governed by the Swedish Parasport Federation to other NSOs. This process unfolds in multiple stages, with the aim of improving conditions for athletes, coaches, and local sports clubs involved in parasport (44).

Participants' roles involved coordinating a steering group that included the organization's Secretary General and Chairperson and maintaining an ongoing dialogue with the Swedish Parasport Federation. In addition, their responsibilities include proactively working with communication and organizational development related to the inclusion process, both internally and externally. The selection of federations was made in consultation between the researchers and the coordinator ultimately responsible for the inclusion process at the Swedish Parasport Federation. The selection was based on the following criteria: (1) all federations included had initiated the inclusion process; (2) the sample included both federations that had progressed further in the process, such as table tennis and swimming, and those at an earlier stage, such as football and floorball. The intention was to include federations representing both individual and team sports, as well as both smaller federations, such as table tennis, and larger ones, such as football.

The interviewees brought diverse expertise to the study. Some had trained or competed at elite levels; most had completed various sports leadership programs, and a few held academic degrees in sports-related disciplines. However, none of the participants had personal experience as para-athletes.

The interviews were conducted by the second author using a semi-structured format, which enabled respondents to articulate their perspectives on inclusion from their leadership positions. This approach facilitated conversations that moved beyond political rhetoric, yielding deeper insights into how and why inclusion was being implemented (45, 46). Each interview lasted between 40 and 60 min and was audio-recorded, transcribed verbatim, and subsequently translated from Swedish to English by the first author after the analysis.

## Analysis

Thematic analysis, a qualitative method used to identify patterns, themes, and categories within data (47–49), was employed to guide the analysis. This approach enabled a rigorous and systematic examination of the data, contributing to the validity and reliability of the study's findings. The analysis was conducted by the first author, drawing on the theoretical lenses of SIT and ableism. The coding framework combined predefined themes informed by previous research (26). Through repeated engagement with the data, the first author identified recurring patterns, points of divergence, and interconnections among themes. Given the centrality of identity in this study, particular attention was paid to how the respondents conceptualized and articulated their identities in their reflections.

The coding process involved the following steps:

1. Familiarization with the transcripts through repeated reading
2. Identifying and labeling text fragments relevant to the research objectives
3. Searching for patterns within and across interviews
4. Defining, naming, and organizing the data for analysis according to the stated themes

After transcription, all interviews were read repeatedly to ensure familiarity with the data. Initial codes were generated inductively and included terms such as “separation between para and mainstream”, “uncertainty about roles”, “pressure from above”, and “inclusive in theory, not in practice”. These codes were then grouped based on similarities and recurring patterns, allowing for the development of broader themes.

Theme development was iterative and interpretive, involving continuous comparisons across data sets. For example, codes related to the privileging of able-bodied norms and the emphasis on ability were synthesized under the theme *social categorization*. Codes related to how individuals define themselves through group membership and how this shapes their perception and interaction were synthesized under the theme *social identification*. Throughout the process, attention was given to latent meanings in the data and how structural and cultural factors influenced stakeholders' interpretations of inclusion.

## Results

The findings are organized thematically in response to the study's research questions. RQ1 was related to one theme, RQ2 was related to two themes, and RQ3 was related to one theme. Table 2 defines the thematic representation of the data.

**Table 2:** SIT themes and their associated research questions.

SIT Theme	Associated Research Question
Social Categorization	RQ2: How do processes of categorization contribute to ingroup/outgroup dynamics and influence inclusion practices?
Social Identification	RQ1: How do stakeholders construct and understand social identities in the context of inclusion?
Social Comparison	RQ2: How do intergroup comparisons shape inclusion dynamics and decision-making within NSOs?
Social Hierarchy	RQ3: How do stakeholders navigate intersecting social identities beyond the able/disabled binary?

### Social categorization

In the context of parasports and mainstream sports, social categorization occurs when key stakeholders classify athletes into distinct groups based on ability status, such as “able-bodied athletes” and “para-athletes”. These categories are often shaped by institutional structures, language, and dominant norms about what constitutes a “real” or “elite” athlete. While such categorization can support the organization of competition and resource allocation, it also reinforces group boundaries and can lead to the construction of essentialized identities. For example, para-athletes are often collectively perceived as less competitive or less capable, regardless of individual performance. These generalizations may foster stereotypes and further marginalize para-athletes within integrated sport systems.

The excerpts below illustrate how able-bodied norms are often privileged while disability is marginalized (28) and how the emphasis on ability can reinforce exclusionary dynamics (29).

I can't even imagine what it means to these people [para-athletes] to be part of a team like this [a professional sports team]. You can see the joy and community they have. Many in this group suffer from both mental and physical illness[es] because they are not as active and do not have a forum where they can meet. They feel quite lonely in society. I get touched when I see it, and I think those in management who witness this understand why we must take care of this group. (501A1)

The language in this quote subtly reinforces a divide between “us” and “them”, positioning para-athletes as inherently vulnerable. This framing focuses on the individual's disability rather than considering the broader environmental or structural barriers to participation. It also risks homogenizing the experiences of PwDs, thus drawing on stereotypical understandings of disability rather than acknowledging the diversity that exists within this group.

However, the data also revealed instances where participants questioned or resisted such binary constructions. One interviewee expressed a more inclusive perspective:

So it is, overall, important to build bridges between people, regardless of whether you have a disability or not, and regardless of whether you were born in Umeå, Rinkeby, Santiago, Chicago, Canberra, Nairobi, or wherever you come from. (504A1)

In 2015, more than 160,000 people sought asylum in Sweden (50). This large-scale influx had a notable impact on the capacity of the Swedish sports movement to facilitate inclusion, and several interviewees referred to this period as their primary point of experience in relation to inclusion and integration efforts. The following excerpt illuminates experiences of inadequate conditions and insufficient preparation for the change to result in effective and sustainable inclusion, as well as the gap between policy and practice (18, 19, 22).

I know, for instance, that when we worked with the refugee influx, people working in the clubs said, “Well, I’m here to coach my own children and their friends. This isn’t something I know how to do.” The willingness is probably there, but the knowledge, the resources, and the capability are definitely not fully in place. (512A1)

### Social identification

According to SIT (26), social identification refers to the process through which individuals define themselves in relation to their membership in social groups. People derive a substantial part of their self-concept by identifying with a group, which in turn shapes how they perceive themselves and interact with others (27). This process underpins inter-group dynamics and often leads to ingroup favoritism and the marginalization of outgroups (37).

Once social categories are established, individuals begin identifying with particular groups, such as members of the able-bodied community or participants in mainstream sports. These affiliations become a salient component of social identity, shaping behavior, values, and expectations. Within mainstream sports, this identification can foster belonging and solidarity, which may be empowering (51). Conversely, identification with a stigmatized or subordinate group—such as para-athletes—can lead to internalized stigma and heightened feelings of exclusion (4). One of the interviewees expressed this in this way:

The recipients are not prepared, either in terms of resources or knowledge, and it becomes a huge challenge when everything is suddenly dumped on us. It sounds very politically correct, and I do believe that we should work toward inclusion, but I see a significant risk in just pushing ahead without preparation. What happens when, all of a sudden, a girl or boy in a wheelchair arrives, and the person dropping them off thinks it's simply a matter of handing them over? Those receiving them have no idea what to do or how to handle the situation. It might go really well, but there's a major challenge in this. (512A1)

Moreover, individuals operating within mainstream sports organizations may adopt and reproduce dominant norms that prioritize able-bodied athleticism, thereby shaping how inclusion is interpreted and enacted. These dynamics underscore the complex role that social identification plays in either reinforcing or challenging structures of exclusion. In the following quote, one interviewee positions themselves and their organization within the “table tennis family”, emphasizing a sense of shared identity and belonging. This reflects self-categorization and a positive ingroup association: “We are heavily engaged in the development of Swedish table tennis, almost like a family. ‘Table tennis for everyone’ is one of our major slogans” (507A1).

This study also aimed to examine how stakeholders navigate intersecting social identities beyond the able/disabled binary and how such navigation influences their approaches to fostering inclusion without reinforcing status hierarchies or ingroup bias. For example, according to one of the interviewees, the inclusion process is an important catalyst to organizational development:

We need to be courageous, and I believe we now have a board that is brave and does not stand for preserving things, just as they have always been. There will be conflicts about this. I'm completely convinced of that, but that's how it is. That's what drives development: engaging in debate and challenging each other's views. (510A1)

Other participants described how accommodations initially introduced for para-athletes—such as the use of Sign Supported Speech (SSS)—also proved useful for other participant groups, including children. Above all, such outside-the-box thinking encouraged creativity and helped to identify new ways to lower the threshold for participation in sports more broadly. This contributed added value from the perspective of organizational development and showed that, in the long term, broadening activities through the inclusion process can attract more participants:

We thought this would be extremely useful for this particular group of individuals, but we quickly realized that all other groups benefited just as much from the clarity and support as the children with Down syndrome. For me, it became clear that, well, this is something everyone actually needs—it's useful for all. So, in reality, you're not making a distinction; it's just that we assumed there was a difference. Of course, adjustments are necessary, but I believe we are constantly adapting to the individual, not to the disability. I think it's a mistake to assume that there are vast differences. (503A1)

### Social comparison

Following categorization and identification, group members engage in social comparison—the process of evaluating their own group (the ingroup) in relation to relevant outgroups in order to maintain a positive collective identity (26, 36). This comparison underpins the pursuit of positive distinctiveness, whereby ingroup characteristics are accentuated and outgroup qualities are downplayed (37, 52).

In sports, these dynamics are often enacted through comparisons between able-bodied and para-athletes or between mainstream and parasport organizations (11, 30). Such comparisons tend to reinforce existing hierarchies: Mainstream sports are portrayed as serious or elite, whereas parasports are often framed as inspirational or niche—seemingly positive labels that nevertheless marginalize para-athletes (8, 24). Recent studies of local sports clubs show that even well-intentioned “benevolent” framings can sustain able-bodied privilege within inclusion efforts (31). These themes also emerged clearly in the interviews, as in the following quote: *“*All the committees talk about ‘healthy table tennis’ and ‘para table tennis,’ just as they talk about youth and veterans. So there are categories, but no sub-groups” (505A1).

Here, the categorical separation of “para table tennis” from “heathy table tennis” implicitly frames the former as ancillary, not integral to the sport. This kind of comparative distinction exemplifies how mainstream status is protected through favorable comparison.

Such framings reveal an implicit ableist logic, wherein disability is constructed as a separate and exceptional category rather than as a natural part of athletic diversity. This perception reflects the underlying assumption that “normal” athletes form a homogeneous group, contributes to the process of “othering”. By treating disability as fundamentally distinct, such assumptions risk reinforcing exclusionary norms within sport.

These findings align with previous research showing that ableism remains deeply embedded in sports culture, where normative ideals of physicality and competition dominate. Studies have demonstrated that ableist structures within sports institutions contribute to unequal opportunities and the marginalization of para-athletes (54). As such, mainstream sports organizations frequently reproduce rather than challenge systemic inequalities.

One commonly cited barrier to implementing inclusion was a lack of knowledge among sports clubs. However, the interviews suggest that this barrier is often framed as a technical issue to be solved through education rather than as a structural power imbalance that requires deeper institutional change. Further, some interviewees emphasized the need for knowledge exchange and collegial learning to bridge the gap between “us” and “them”. However, even in these reflections, the divide remains evident. One participant offered the following reflection:

It easily becomes a side business. When I didn't know anything about this, I distanced myself out of fear of making a mistake. We need an expert center, similar to how the Swedish Football Federation has experts on gender equality or honor-related violence. We must digitize knowledge sharing, create forums for experience exchange, and actively engage with the target group. (501A1)

### Social hierarchy

Within SIT, *social comparison* frequently results in the status-ordering of groups. When one group consistently enjoys greater prestige, access to resources, and cultural legitimacy than another, a social hierarchy is established and, unless actively contested, reproduced (26, 52). In sports, this hierarchy is reinforced by institutional practices (e.g., media coverage, funding criteria, competition calendars) that privilege able-bodied performance while relegating parasports to a subordinate tier (8, 11).

From a critical disability studies perspective, such rankings reflect *ableism*, an ideological system that locates “normal” athleticism in able bodies and frames disability as deviation (28). Social-dominance theory further predicts that high-status groups will defend these arrangements to maintain both symbolic and material advantages and material advantage (53). Some interviewees pointed to a clear hierarchy between parasports that have a natural home within an NSO and those that lack such support entirely, such as boccia and showdown.

Good inclusion means that the sport belongs where it naturally fits/…/ that table tennis is played within the table tennis federation, and swimming takes place within the swimming federation./…/ You can't force a federation to take on boccia or showdown. It's going to be really difficult. It won't work if there's no receiving body that actually wants to take them on. (507A1)

This excerpt reflects an implicit ranking of sports, where those that are perceived as having a “natural” organizational home are deemed more legitimate than those that do not. Sports without a mainstream equivalent, such as boccia and showdown, appear to be positioned lower in this hierarchy, suggesting that perceived structural fit may serve as a rationale for their marginal status.

Another interviewee highlighted how the interplay of multiple marginalized identities can exacerbate exclusion, illustrating the principle of *cumulative disadvantage*. This concept underscores that social hierarchies are not merely additive but intersectional, as overlapping dimensions of inequality intensify structural barriers [see (31)].

Sport has very strong norms regarding how one is expected to behave. So, if a person with a disability also deviates from the sport norm due to, for instance, skin color, socioeconomic status, age, or sexual orientation, it becomes twice as difficult for that individual. (509A1)

There were also examples of clear resistance to social hierarchy, including the following:

The children with Down syndrome who play floorball—of course, they're here playing floorball just like we all do. They shouldn't be placed somewhere else, separated and invisible. That, I think, is the real gain of this [inclusion process]. (510A1)

In its condemnation of exclusion, this quotation reveals how physical separation operates as a hierarchical practice that diminishes the status of PwDs and calls for the removal of status differentials. In the next example, the interviewee challenges the linguistic distinctions that sustain social hierarchies, emphasizing that the lower-status group (parasport participants) is heterogeneous, thereby resisting the homogenizing logic that underpins exclusion:

I feel that the terminology is problematic—it often results in an ‘us and them’ narrative. However, those involved in parasport also come from diverse socioeconomic backgrounds and ethnicities and have varying levels of ambition and different interests, just like other members. (511A1)

As seen in previous examples, several interviewees used the term “family”, which may suggest that inclusion has either been achieved or, at the very least, is experienced positively. The following quotation offers a counter-hierarchical perspective. By invoking the “family” metaphor, the interviewee repositions para-athletes as equal members within a unified sporting community:

We are very proud that we were early in including para-athletes in our activities/…/ I really like the idea of “one sport, one family” because in the clubs and federations, we are together. (505A1)

In the following example, the interviewee explicitly advocated for structural equality by implicitly critiquing the existing multitiered system in which separate NSOs signify unequal status. It further illustrates efforts to challenge the hierarchy between able-bodied and disabled participants: *“*Of course, everyone who plays football, regardless of their individual circumstances, should reasonably be doing so within the framework of a single football federation” (502A1).

Collectively, these excerpts demonstrate that stakeholders not only recognize entrenched hierarchies in sports but also articulate both the mechanisms that sustain them and the counter-discourses that seek to dismantle them.

## Discussion and conclusion

This study applied SIT and the concept of ableism to explore how key stakeholders within NSOs construct, perceive, and navigate social identities in the context of inclusion across parasports and mainstream sports. It explored how processes of social categorization, identification, and comparison contribute to the emergence of ingroup/outgroup dynamics—particularly between able-bodied and disabled identities—and how these dynamics shape inclusion practices, intergroup relations, and the broader pursuit of social change. Additionally, the study investigated how stakeholders navigate intersecting social identities beyond the able/disabled binary and how these intersections influence their strategies for fostering inclusive environments without reinforcing existing power relations, hierarchies or ingroup bias.

The findings show that current inclusion efforts remain primarily focused on PwDs rather than all athletes. This narrow scope may limit the broader transformative potential of inclusion. Two overarching perspectives on inclusion emerged. The first maintains a clear division between “us” and “them”, often predicated on the assumption that sports inherently benefit PwDs, who are at times framed as passive recipients of care. This view risks oversimplifying the diverse experiences and aspirations of para-athletes by treating them as a homogeneous group. The second perspective challenges normative assumptions about sports by emphasizing the value of including individuals from diverse backgrounds as active contributors to the evolution of sports at all levels within the Swedish Sports Confederation. Here, inclusion is not merely about access but also about meaningful representation and participation in decision-making processes—changes that are perceived as beneficial for the sports community as a whole. Several interviewees suggested that increasing participation and representation of para-athletes could generate broader improvements across sport systems.

A central challenge remains the need for research that goes beyond identifying barriers to inclusion and instead drives structural transformation in the lives of PwDs. This study indicates that sports organizations often expect para-athletes to conform to existing norms—both physical and cultural—without critically interrogating those norms or considering how they might be reimagined. Yet fostering a more inclusive environment in which athletes interact as equals is consistent with the democratic values expressed in the Swedish Sports Confederation's policy documents. Achieving such change requires decision-makers to balance equal treatment with individualized support and to implement environmental changes that promote accessibility. It also demands that they acknowledge their own influence in either reinforcing exclusion or enabling transformation.

Like all research, this study has limitations. The data were collected shortly after the COVID-19 pandemic, and participants' views may have evolved since then. One contributing factor may be the practical difficulties of advancing inclusion during a time marked by extensive social restrictions and isolation. For para-athletes in particular, the pandemic exacerbated existing barriers to participation, as many inclusive initiatives were postponed or deprioritized due to health concerns and limited access to training environments. These exceptional circumstances likely shaped how inclusion was perceived and practiced at the time of the interviews. However, as regulatory and policy frameworks have remained relatively stable, the findings still offer a valid account of stakeholder perspectives.

These findings invite further reflection on what meaningful inclusion in sport entails. Future research could investigate effective models and practices that have successfully created accessible and inclusive sporting environments. Rather than relying on symbolic gestures or surface-level interventions, genuine inclusion appears to require sustained, targeted efforts that address underlying structural inequalities. These include fostering team cultures built on mutual respect, promoting inclusive leadership, and ensuring that organizational practices reflect fairness and accessibility. When approached in this way, sports can transcend a mere site of performance to become a platform for empowerment, belonging, and transformative social change.

For inclusion to have a lasting impact, it must be fully integrated into the core practices of sports organizations. Isolated or short-term initiatives are unlikely to lead to sustainable outcomes. Long-term success requires that emerging challenges be met through a consistent translation of organizational values into everyday actions. This, in turn, necessitates the engagement of all key actors—coaches, board members, athletes, and others involved in sports—in a shared process of change. Such commitment entails a collective vision and a willingness within Swedish sports to pursue meaningful and enduring change through organizational development, education, communication, and critical examination of the normative assumptions that define sports.

Finally, collaboration among researchers, para-athletes, sports organizations, and policymakers is essential for translating research into meaningful practice. This study demonstrates how stakeholder expectations and assumptions can both challenge and reproduce ableist norms. Many of the interviewees' reflections reveal a process of “othering”, portraying para-athletes as a group in need of special support rather than identifying the systemic barriers to participation. The continued positioning of mainstream sports as the normative ideal highlights the importance of critically addressing ableism in future inclusion initiatives.

## Data Availability

The raw data supporting the conclusions of this article will be made available by the authors, without undue reservation.
